# Impairment of Neuronal Glutamate Uptake and Modulation of the Glutamate Transporter GLT-1 Induced by Retinal Ischemia

**DOI:** 10.1371/journal.pone.0069250

**Published:** 2013-08-06

**Authors:** Rossella Russo, Federica Cavaliere, Giuseppe Pasquale Varano, Marco Milanese, Annagrazia Adornetto, Carlo Nucci, Giambattista Bonanno, Luigi Antonio Morrone, Maria Tiziana Corasaniti, Giacinto Bagetta

**Affiliations:** 1 Department of Pharmacy, Health and Nutritional Sciences, University of Calabria, Arcavacata di Rende, Italy; 2 University Consortium for Adaptive Disorders and Head Pain (UCHAD), Section of Neuropharmacology of Normal and Pathological Neuronal Plasticity, University of Calabria, Arcavacata di Rende, Italy; 3 Department of Pharmacy, Unit of Pharmacology and Toxicology and Center of Excellence for Biomedical Research, University of Genoa, Genoa, Italy; 4 Ophthalmology Unit, Department of Experimental Medicine and Surgery, University of Rome “Tor Vergata,” Rome, Italy; 5 Department of Health Sciences, University “Magna Graecia” of Catanzaro, Catanzaro, Italy; Dalhousie University, Canada

## Abstract

Excitotoxicity has been implicated in the retinal neuronal loss in several ocular pathologies including glaucoma. Dysfunction of Excitatory Amino Acid Transporters is often a key component of the cascade leading to excitotoxic cell death. In the retina, glutamate transport is mainly operated by the glial glutamate transporter GLAST and the neuronal transporter GLT-1. In this study we evaluated the expression of GLAST and GLT-1 in a rat model of acute glaucoma based on the transient increase of intraocular pressure (IOP) and characterized by high glutamate levels during the reperfusion that follows the ischemic event associated with raised IOP. No changes were reported in GLAST expression while, at neuronal level, a reduction of glutamate uptake and of transporter reversal-mediated glutamate release was observed in isolated retinal synaptosomes. This was accompanied by modulation of GLT-1 expression leading to the reduction of the canonical 65 kDa form and upregulation of a GLT-1-related 38 kDa protein. These results support a role for neuronal transporters in glutamate accumulation observed in the retina following an ischemic event and suggest the presence of a GLT-1 neuronal new alternative splice variant, induced in response to the detrimental stimulus.

## Introduction

L-glutamate is the major excitatory neurotransmitter in the Central Nervous System including the retina, where it is released by photoreceptors, bipolar and ganglion cells [1], [2] and is responsible for the transmission of the light signal. The physiologic concentration of glutamate at the synaptic cleft is maintained by Na^+^-dependent, high-affinity transporters identified as Excitatory Amino Acid Transporters (EAATs), which are located on both neurons and glia [3]. In the retina, four out of the five known EAATs have been described: EAAT1 (also known as GLAST) expressed by Mϋller cells; EAAT2 (glutamate transporter-1; GLT-1) localized on photoreceptors and bipolar cells; EAAT3 (EAAC1) detected in horizontal, ganglion and some amacrine cells; EAAT5 is associated with photoreceptors and bipolar cells [4], [5].

Besides its role as neurotransmitter, glutamate is also a potent neurotoxin [6], [7], therefore the efficiency of glutamate transporters is crucial not only to terminate the excitatory signal, but also to prevent the excitotoxic neuronal damage [8]–[10].

Many experimental evidence suggest that excitotoxicity is one of the main factors involved in ganglion cell death observed during retinal hypoxic/ischemic events [11]–[14] which are common in several ocular pathologies including diabetic retinopathy, retinal and choroidal vessels occlusion and glaucoma [15]–[17]. This hypothesis is strongly supported by the neuroprotection afforded by intravitreal or systemic treatment with NMDA and non-NMDA receptor antagonists [11], [13], [18], [19] or by the open channel blocker memantine [20], [21] in acute and chronic models of retinal ganglion cells (RGCs) death.

As for other neurodegenerative disorders characterized by excitotoxic events, dysfunction of glutamate transporters has been found as part of the cascade leading to retinal neuronal death under different experimental and clinical pathological conditions [22], [23]. However, the role of EAATs in retinal injuries, and in particular under retinal ischemia/reperfusion, remains controversial [24]–[26]. Most of the available data are related to the ischemic phase of retinal injury, while less is known on the role of EAATs during the reperfusion phase, which is crucial for the damage propagation and therefore the extent of neuronal death. Furthermore, due to their relevance in glutamate clearance, several studies focused on glial glutamate transporters while fewer information have been gained on the role of neuronal glutamate transporters.

Aim of this study was to further explore the function of EAATs under ischemic retinal conditions, and to extend our knowledge on their role during the following reperfusion phase. To this end, we examined the expression of GLAST and GLT-1 in a model of acute retinal ischemia induced by transient increase of IOP and characterized by high glutamate levels during the reperfusion phase [27].

## Results

### GLT-1 and GLAST modulation under retinal ischemia/reperfusion

We have previously reported a significant increase of vitreal glutamate in the ischemic retina that peaks after 150 min of reperfusion [27]. To investigate whether or not this event was associated with a modulation of glutamate transporters, the distribution of the two most abundant EAATs in the retina, i.e. GLAST and GLT-1 [33]–[35], has been evaluated by immunofluorescence.

**Figure 1:**
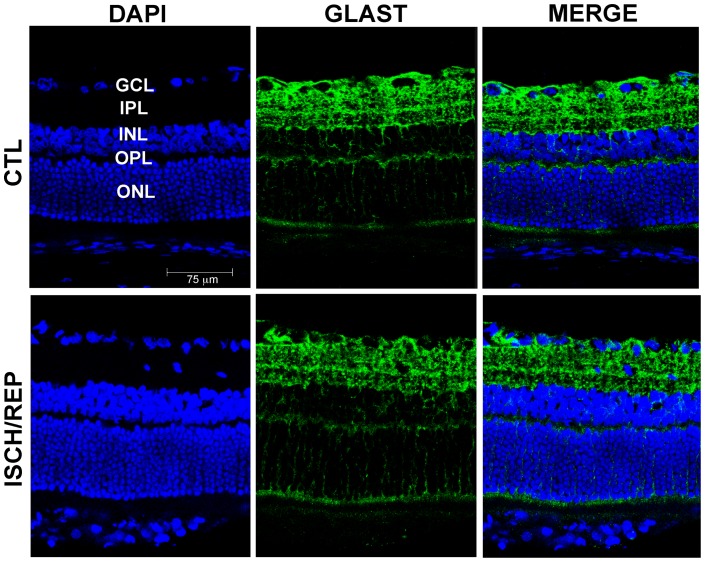
Representative immunofluorescence of retinal sections showing the expression pattern of GLAST following ischemia. Glast immunoreactivity in the ischemic retina after 150 min of reperfusion (ISCH/REP) shows no evident changes in the expression and distribution of the glial transporter compared to the contralateral non-ischemic retina (CTL). (GCL) ganglion cell layer (IPL), inner plexiform layer, (INL) inner nuclear layer, (OPL) outer plexiform layer, (ONL) outer nuclear layer.

**Figure 2:**
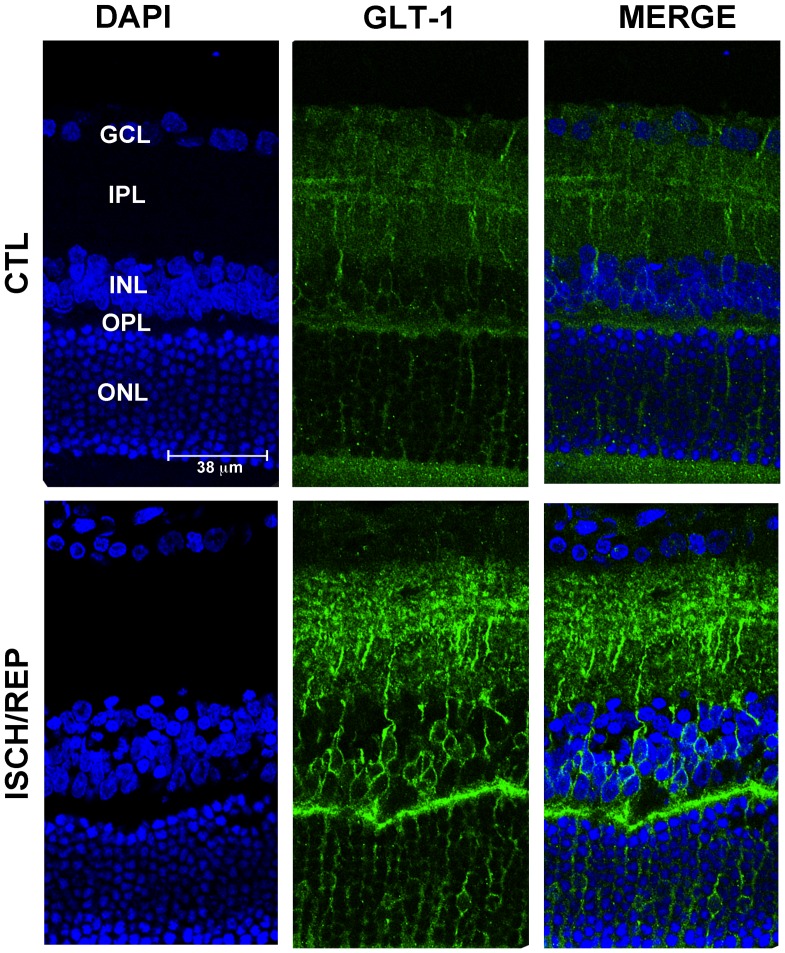
Effects of retinal ischemia/reperfusion on the expression and distribution of glutamate transporter GLT-1. GLT-1 immunoreactivity increases in the retina of rats subjected to 50 min of ischemia followed by 150 min of reperfusion (ISCH/REP) as compared to the control, non-ischemic retina (CTL). An increased number of GLT-1 positive cell bodies is evident in the ONL and INL of retinas subjected to ischemia (ISCH/REP). (GCL) ganglion cell layer (IPL), inner plexiform layer, (INL) inner nuclear layer, (OPL) outer plexiform layer, (ONL) outer nuclear layer.

In the control retina, GLAST immunoreactivity was diffused from the outer to the inner limiting membrane (Figure 1, CTL) and no changes in its expression were detected in the ischemic retina after 150 min of reperfusion (Figure 1, ISCH/REP). It is established that retinal GLAST expression is limited to astrocytes and Mϋller cells whereas GLT-1 is found in neurons, mainly on photoreceptors and various types of bipolar cells [36], [37]. In agreement with this distribution, here GLT-1 was expressed in bipolar cells bodies of the inner nuclear layer (INL) and in bipolar cells processes and photoreceptors terminals at the inner and outer plexiform layers (IPL, OPL) under control conditions (Figure 2, CTL).

The pattern of GLT-1 expression was increased after ischemia followed by 150 min of reperfusion (Figure 2, ISCH/REP) when compared to the contralateral eye (Figure 2, CTL) and this effect is at variance with the unchanged GLAST expression. A stronger GLT-1 immunoreactivity was detected both in cell bodies (INL) and neuropils (IPL) of bipolar cells, as well as at synaptic contacts between bipolar and ganglion cells (IPL) (Figure 2, ISCH/REP). Moreover, an increased number of GLT-1 positive cell bodies was evident in the outer nuclear layer (ONL) and INL (Figure 2, ISCH/REP).

### Retinal ischemia decreases [^3^H]-D-Aspartate uptake

**Figure 3:**
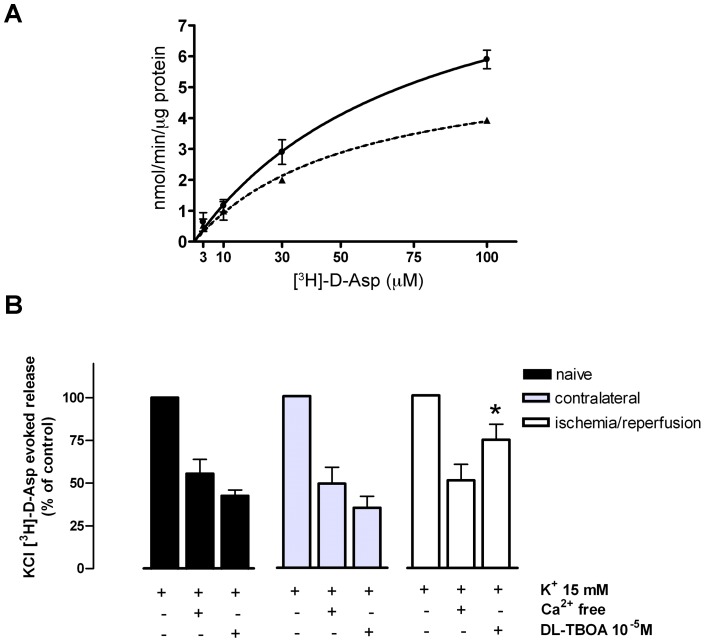
Modulation of [^3^H]-D-Aspartate release and uptake in synaptosomes from ischemic retina. (A) *Ischemia reduced [^3^H]-D-Asp uptake in highly pure nerve ending.* [^3^H]-D-Asp uptake into synaptosomes was measured in animals subjected to retinal ischemia for 50 min in the right eye and reperfused for 150 min (dashed line). The contralateral eye (solid line) was used as control. Data are expressed as mean ± S.E.M. (n = 3 or 4 animals per group). (B) *[^3^H]-D-Asp release in retinal synaptosomes.* Animals were subjected to retinal ischemia for 50 min in the right eye and reperfusion was allowed for 150 min. Release was measured in the ischemic (white histograms), contralateral (grey) and naïve retinas (black). All results are expressed as percentage of the corresponding KCl-evoked release (15 mM). Omission of Ca^2+^ produces a reduction of [^3^H]-D-Asp release of about 40% in all groups, while DL-TBOA reduces by about 60% the release in naïve and contralateral samples, but not in the ischemic retina. *p<0.05 vs control.

In order to evaluate if the increased GLT-1 immunoreactivity was paralleled by a modulation of the neuronal transporters activity, we isolated the retinal synaptic terminals (synaptosomes) and performed uptake experiments using the non-metabolizable glutamate analogue [^3^H]-D-Aspartate ([^3^H]-D-Asp). As shown in Figure 3A the apparent Km value for [^3^H]-D-Asp uptake did not significantly change between ischemic (39.24±4.49 µM) and contralateral synaptosomes (48.44±6.12 µM). However, a significant difference could be detected for the Vmax value (6.04±0.32 and 10.42±0.40 nmol/min/μg protein in ischemic and contralateral retina, respectively; p<0.05) leading to a 42% reduction of [^3^H]-D-Asp uptake in the ischemic synaptosomes.

### Ischemia/reperfusion reduces transporter-mediated [^3^H]-D-Aspartate release in retinal synaptosomes

To further characterize the functional role of GLT-1 in the ischemic retina, we performed release experiments aimed to dissect the different mechanisms underlying the depolarization-evoked [^3^H]-D-Asp release. Synaptosomes pre-labeled with [^3^H]-D-Asp were depolarized by adding KCl (15 mM). In this experimental condition, the tritium release evoked by KCl in the naïve retina was 2.58±0.56% of the total tritium content; total transmitter release in the contralateral and ischemic eyes did not vary significantly compared to naïve eye being 2.85±0.52% and 3.03±0.32%, respectively. However, the mechanism underlying the K^+^-evoked [^3^H]-D-Asp release was different between control and ischemic synaptosomes. In particular, while the Ca^2+^-dependent release of [^3^H]-D-Asp accounted for 40% of the total release in all groups (Figure 3B), the percentage of the transporter-mediated release differed between groups. In fact, exposure of synaptosomes from non-ischemic retinas to the glutamate transporters inhibitor DL-TBOA (100 µM) reduced the evoked-release of [^3^H]-D-Asp by 60%, while this was reduced by only 26% in the ischemic synaptosomes.

### Ischemia/reperfusion reduced the levels of mature GLT-1

**Figure 4:**
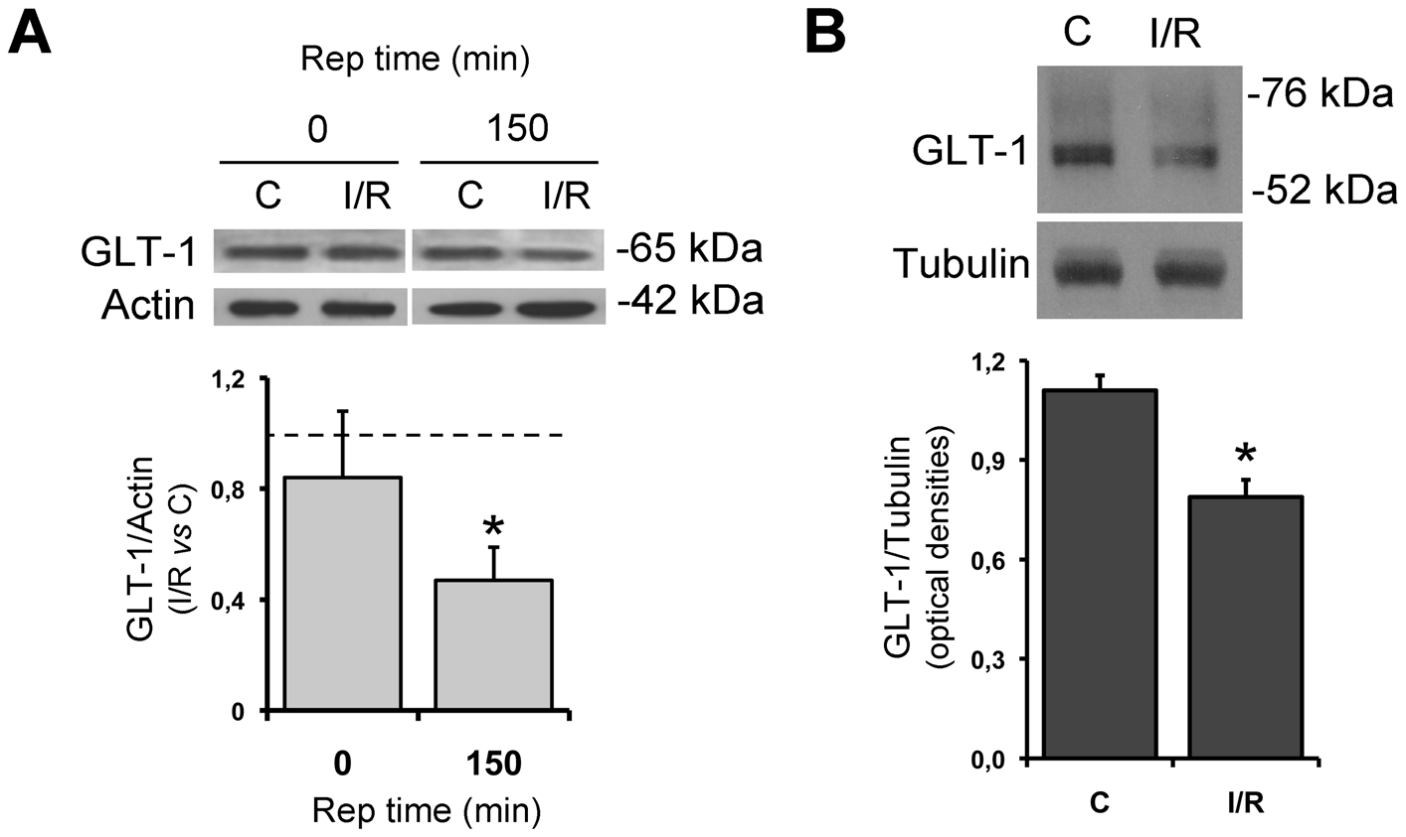
Reduction of GLT-1 in synaptosomal fraction (A) and total extracts (B) from ischemic retinas. Animals were subjected to retinal ischemia in the right eye (I/R) and left eye (C) was used as control. (A) In the synaptosomal fraction experiments reperfusion was allowed for 0 and 150 min and the results expressed as fold changes of the I/R vs C. (B) Immunoblot showing the changes of GLT-1 expression in the total retinal extract after 150 min reperfusion. Histograms represent densitometric analysis of immunoreactive bands from three independent experiments. *p<0.05 vs contralateral samples.

The lower Vmax value detected for the ischemic retinas in the uptake studies and the reduced transporter reversal-mediated release of [^3^H]-D-Asp could be due to an altered activity and/or a reduced expression of the neuronal transporters at the synaptic buttons. To address this point we performed immunoblotting experiments with purified synaptosomes that revealed a 16% decrease of expression of the 65 kDa band, corresponding to the mature GLT-1, at the end of ischemia and a more pronounced and statistically significant reduction (52%) after 150 minutes of reperfusion (Figure 4A). Interestingly, at this same time point, a significant reduction of GLT-1 expression, was observed also in total retinal extracts from ischemic eye when compared to the contralateral eye (Figure 4B).

These data mirror the decreased Vmax reported in the uptake experiments and the reduced transporter-mediated component in the release experiments but did not support the previously observed GLT-1 immunoreactivity increase detected by immunofluorescence (Figure 2).

### Retinal increase of GLT-1 is dependent on new protein synthesis

To rule out the possibility that a technical artifact might account for the discrepancy between biochemical and immunohistochemical data we evaluated if the increased GLT-1 immunoreactivity under ischemic condition was sensitive to pharmacological modulation.

**Figure 5:**
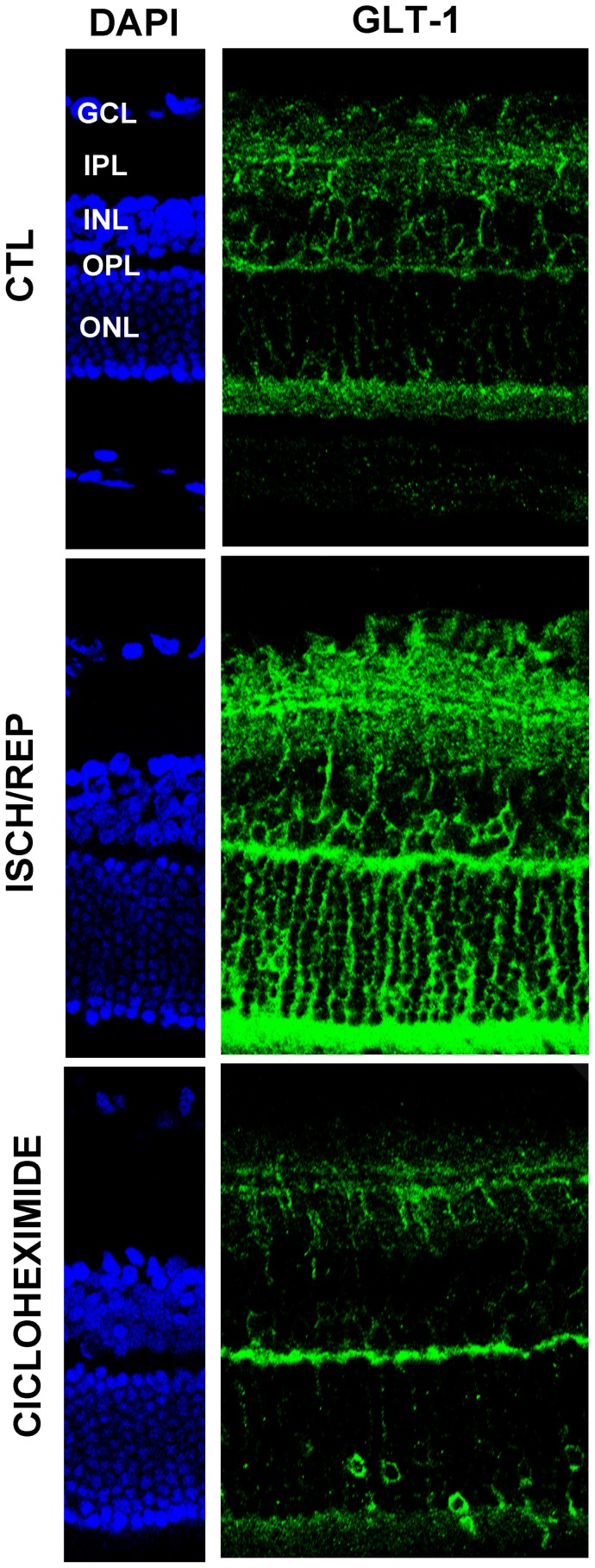
Effect of protein synthesis inhibition on GLT-1 increased immunoreactivity induced by retinal ischemia. Intravitreal administration of the protein synthesis inhibitor cycloheximide, at the end of the ischemia, prevents GLT-1 immunoreactivity increase at 150 min of reperfusion (ISCH/REP). Images are representative of three independent experiments. (CTL =  non-ischemic contralateral eye).

Intravitreal administration of the protein synthesis inhibitor cycloheximide (CHX; 50 micrograms/eye), at the end of the ischemic period, prevented the increase of GLT-1 immunoreactivity observed at 150 min of reperfusion (Figure 5). This finding confirms the genuine nature of the transporter upregulation detected by immunofluorescence and demonstrates that GLT-1 synthesis occurs during the reperfusion phase.

### GLT-1 expression increased under native conditions

**Figure 6:**
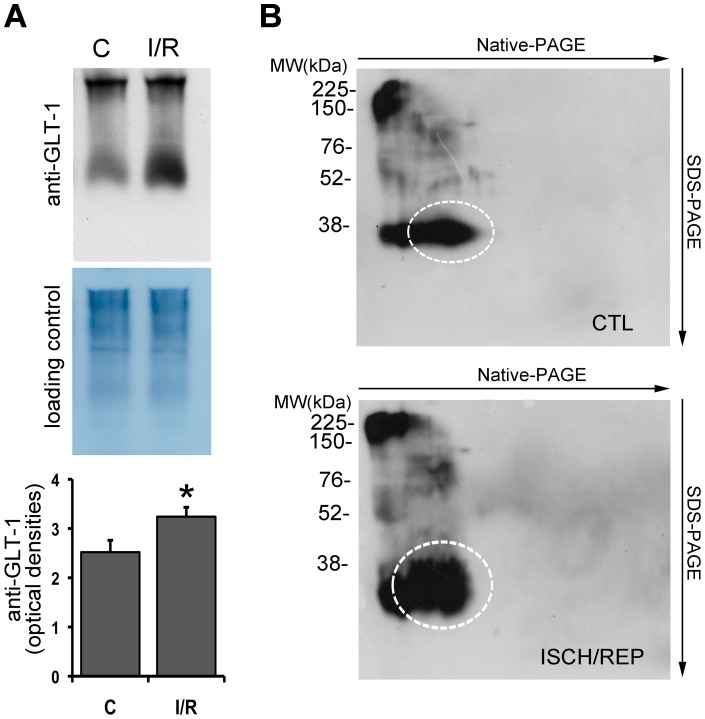
Native and bi-dimensional western blotting analysis of GLT-1. (A) GLT-1 immunoreactivity is increased under native conditions in the ischemic retina (I/R) after 150 min of reperfusion when compared to the non-ischemic contralateral eye (C). Histogram shows the results of the densitometric analysis of autoradiographic bands normalized to the value of loading control. Each value is the mean ± S.E.M. of three experiments. (B) A representative Native/SDS-PAGE image showing the appearance in the second dimension (SDS-PAGE) of a 38 kDa band up-regulated in the retina subjected to 50 min of ischemia and 150 min reperfusion (ISCH/REP) as compared to the contralateral non-ischemic retina (CTL).

On the basis of the latter results, it can be hypothesized that the discrepancy alluded to above could rise from the GLT-1 protein state in the two experimental settings (native in immunofluorescence and denatured in western blotting) and consequently from the ability of the used antibody to recognize the same epitopes. Therefore, we analyzed the total retinal extracts by western blotting under native conditions. The result obtained was consistent with the immunofluorescence data showing a significant increase of GLT-1 immunoreactivity after 150 min of reperfusion when compared to non-ischemic eye (Figure 6A).

In order to resolve the native complex, this was separated in a second dimension under denaturing and reducing conditions. Surprisingly, in the complex we detected the presence of a protein with an immunoreactive molecular weight of approximately 38 kDa that was significantly upregulated in the ischemic retina, while only weak immunoreactivity was reported for the band at 65 kDa, given as the mature GLT-1 (Figure 6B).

## Discussion

Elevation of extracellular glutamate is a key factor in retinal neurodegeneration occurring in glaucoma and other retinal pathologies characterized by ischemic events [12], [13], [38], [39]. The activity of sodium-dependent high affinity EAATs is the primary mechanism to maintain glutamate homeostasis and alterations of their expression and function have been found in several neurological disorders [22], [23]. Here we studied the modulation of two out of five EAATs present in the retina, i.e. GLAST and GLT-1 during retinal ischemia/reperfusion, showing a decrease of neuronal glutamate uptake associated with a significant modulation of GLT-1 while no significant changes of GLAST expression were evident.

GLAST, expressed by Mϋller cells [34], [40], is the predominant glutamate transporter in the retina and it is primarily responsible for uptake under physiological conditions [5], [41]. In our experimental system, we reported no changes in GLAST expression and distribution during the reperfusion phase. This result is in agreement with previous experimental observations that showed no modulation of GLAST after ischemia induced by optic nerve ligation [33], following IOP increase by laser photocoagulation of trabecular meshwork [42] or episcleral vein cauterization [43]. Previous work suggests that GLAST function is compromised during retinal ischemia but it is regained during reperfusion [33] even though the transporter saturates at lower glutamate concentrations compared to physiological conditions [44]. Altogether, these data suggest that alterations of glial transporters would, if any, only partially contribute to the glutamate increase that we have previously reported following an ischemic event induced by transient elevation of IOP [13], [27].

At variance with the latter conclusion, our present functional data on glutamate uptake in retinal synaptosomes showed a significant reduction of glutamate transport in the nerve terminals under ischemia/reperfusion suggesting that the failure of neuronal transporters may be a key component in the accumulation of extracellular glutamate observed *in vivo* [27]. In contrast to other areas of the brain, GLT-1 (EAAT2) is expressed in the retina only by neurons and several studies have pointed out a role for this transporter subtype in the pathology of neurodegenerative diseases including glaucoma [42], [43]. Moreover, the presence of GLT-1 on bipolar cells near their synapses with RGCs suggests that GLT-1 activity may be crucial in regulating glutamate concentration around RGCs (24), the cellular subtype known to degenerate during glaucoma [45].

Paralleling the above functional studies, we also observed a reduction of GLT-1 in total extracts and in synaptosomal fraction from retinas subjected to ischemia followed by reperfusion. Again, this may account for the altered glutamate clearance at the synaptic cleft during reperfusion and, therefore, for the excitotoxic retinal neuronal death even when GLAST expression is unchanged.

This hypothesis is supported by the evidence that in rat treatment with antisense oligonucleotides against GLT-1 increases vitreal glutamate levels leading to ganglion cell death [10]. Likewise, retinal damage following ischemia is exacerbated in GLT-1 deficient mice, though the effect is milder when compared to GLAST knockdown [40].

The reduction of GLT-1 expression observed in our study is in agreement with data previously reported in other experimental models of glaucoma. Indeed, a decrease of GLT-1 was found after trabecular laser treatment [24] and in transgenic mice bearing spontaneous ocular hypertension [26]. *Vice versa*, GLT-1 was increased in photoreceptors and bipolar cells from eye subjected to episcleral vein cauterization [43]. GLT-1 down-regulation and consequent glutamate increase have also been reported following focal cerebral ischemia in the rat cortex [46] and global ischemia in astrocytes derived from hippocampus [47]. The differential regulation of this transporter reported under different stress conditions would suggest that GLT-1 regulation is strictly dependent on the neuronal area affected as well as on the type of detrimental stimulus applied.

Although the immunoblotting and functional data reported here concord with a decrease of GLT-1 expression, immunohistochemistry experiments reported an opposite trend, clearly showing a protein synthesis-dependent over-expression of GLT-1 after ischemia/reperfusion. In the insulted retina, controversial outcomes have often been reported using different analytical methods to test the expression of glutamate transporters. For instance, Martin and colleagues described a reduction of GLT-1 by immunoblotting but no changes by immunohistochemistry [24]. These investigators ascribed the conflicting findings to limitations of the methodologies. However, the latter conclusion does not explain satisfactorily our conflicting data, due to a number of reasons. In fact, the GLT-1 localization we reported here is consistent with its cellular distribution as previously described in the retina [34], [40]. Moreover, modulation of the increased GLT-1 immunoreactivity by CHX, an inhibitor of protein synthesis, supports the hypothesis of an authentic upregulation of GLT-1 at the translational level. The explanation for the discrepancy between the results obtained with the two technical approaches might lie in the biophysical state of the target protein (e.g. native in the immunohistochemistry and denatured in the immunoblotting) and, therefore, in the ability of the antibody to recognize it. Indeed, when the electrophoretic separation was performed under native conditions, we did observe an increase of density of the GLT-1 immunoreactive band, according to the results obtained in immunohistochemistry and at variance with immunoblots performed under denaturing conditions. Increase of GLT-1 expression has been shown following hypoxia [48] or over-activation of NMDA receptors [49], either conditions occur in our experimental model [50], [51]. However, the increased GLT-1 immunoreactivity reported under native condition is not the consequence of an upregulation of the mature GLT-1 protein but, rather, of a GLT-1 related protein with an approximately 38 kDa molecular weight, as evidenced by bi-dimensional gel electrophoresis.

The full length GLT-1 (often referred to as GLT-1a) has an approximate molecular weight, following glycosylation, of about 62 kDa [52]. Together with the original form, several post-transcriptionally regulated isoforms with different molecular weight have been described, including alternative splicing producing different N- and C-termini [53]–[55] and exon-skipping splice variants [56]–[58]. The expression of some of these variants is induced in response to selective cell injuries and alteration of the GLT-1 isoforms relative expression has been reported in some neurodegenerative diseases [59]. The functional variant referred to as GLT-1c is abundant in the retina and is expressed, under physiological conditions, only in photoreceptors [60]. However, its expression pattern changes in human and experimental glaucoma; in the latter, it is expressed also in RGCs [42]. Some isoforms appear to differ for their subcellular localization as well; for instance GLT-1b, unlike GLT-1 which is mainly detected in the membrane of astrocytes, is detected in neurons and astrocytes cytoplasm [61], [62]. Our results showed that, following ischemia, GLT-1 immunoreactivity increases mainly in the perinuclear area of bipolar cells and along their processes. This suggests that, following the reduction of the original GLT-1 levels, there is a compensatory mechanism triggered by hypoxia, glutamate or by other factors inducing the expression of an alternative GLT-1 splicing form that accumulates mainly in neuronal soma.

In conclusion, our data support a role for neuronal transporters in the glutamate accumulation observed in the retina following an ischemic event and suggest the presence of a GLT-1 neuronal new alternative splice variant, which is probably induced as a tentative compensatory mechanism in response to the detrimental stimulus. Likewise for other splice variants already described in the literature, we are unable to speculate on the function or the ability of this 38kDa isoform to generate functional transporters; therefore, further experiments will be needed in order to address these questions.

## Materials and Methods

### Ethics statement

Animal care and experimental procedures were carried out in accordance with the guidelines of the Italian Ministry of Health (DM 116/1992). The protocol (Protocol Number 110000351) was dealt with for the ethical and animal care aspects and approved by the Committee set by the Ministry of Health at the National Institute of Health (Rome). All surgical procedures were performed under deep anesthesia and all efforts were made to minimize suffering.

### Retinal ischemia injury

Adult male Wistar rats (280–330 g) were purchased from Charles River (Lecco, Italy). Animals were housed under a 12 h light–dark cycle with *ad libitum* access to food and water. Retinal ischemia was induced by acutely increasing the IOP as previously described [18]. Animals were deeply anesthetized by intraperitoneal injection of chloral hydrate (400 mg/Kg) and laid on a heating pad to maintain the body temperature at 37°C. Topical anesthesia was induced by 0.4% oxibuprocain eye drops (Novesina, Novartis, Varese, Italy). A 27-gauge infusion needle, connected to a 500 ml bottle of sterile saline, was inserted in the anterior chamber of the right eye, and the saline container was elevated to produce an IOP of 120 mmHg for 50 min. Retinal ischemia was confirmed by whitening of the *fundus*. For each animal, the left eye was used as non-ischemic control. Body temperature was monitored before, during and after ischemia, and animals with value lower than 35.5°C were excluded. The animals were sacrificed by cervical dislocation at the end of the ischemia or at 150 min of reperfusion. Both eyes were immediately enucleated, retinas dissected and processed as described below.

### Preparation of synaptosomes

Synaptosomes were prepared as previously described [28], [29]. The retina was homogenized in 10 volumes of 0.32 mol/L sucrose, buffered at pH 7.4 with Tris–HCl, using a glass-teflon tissue grinder (clearance 0.25 mm, 12 up-down strokes in about 1 min); the homogenate was centrifuged (5 min, 1000 g at 4°C) to remove nuclei and debris, the supernatant was gently stratified on a discontinuous Percoll gradient (2%, 6%, 10%, and 20% v/v in Tris-buffered sucrose) and centrifuged at 33500 g for 5 min. The layer between 10% and 20% Percoll (synaptosomal fraction) was collected, washed by centrifugation and resuspended in a physiological medium with the following composition (mmol/L): Tris 5, Hepes 10, NaCl 140, glucose 10, KCl 2.5, MgCl_2_ 1.2, K_2_HPO_4_ 1.2, pH 7.4. All the above procedures were performed at 0–4°C.

### Uptake experiments

Purified synaptosomes were resuspended in standard medium and suspension aliquots (500 µL) containing 6–9 micrograms of protein were incubated with [^3^H]-D-Asp (3-10-30-100 µM) for 2 minutes at 37 °C. Each sample was washed three times and filtered through Whatman microporous membranes (GF/B) (Millipore, Billerica, MA, USA). Unspecific [^3^H]-D-Asp uptake was obtained by performing the same procedure on parallel samples kept in a bath of water and ice while the glutamate transporters were blocked with DL-threo-beta-benzyloxyaspartic acid (DL-TBOA) (10^−5^ M). The radioactivity on filters was counted in an LKB 1214 Rackbeta liquid scintillation counter.

For [^3^H]-D-Asp uptake experiments, Km and Vmax were calculated by fitting data with the Michaelis–Menten equation built into GraphPad Prism Version 4.0a for Windows (GraphPad Software). Fitted values were compared using the t-test.

### Release experiments

Synaptosomes were incubated at 37°C for 15 min with 0.1 µmol/L [^3^H]-D-Asp. After incubation, aliquots of the suspension (about 10 µg protein) were layered on microporous filters placed at the bottom of parallel superfusion chambers (Superfusion System, Ugo Basile, Comerio, Varese, Italy) maintained at 37°C and superfused with standard medium at a rate of 0.5 mL/min [30]. In order to equilibrate the system, fractions were superfused for 36 min and then collected as follows: two 3 min samples (t = 36–39 and 45–48 min; basal release) before and after one 6 min sample (t = 39–45 min; K^+^-evoked release). A 90s period of depolarization, by exposure to KCl 15 mmol/L, substituting for an isosmotic NaCl concentration, was applied at t = 39 min. When appropriate, DL-TBOA was added 9 min before KCl; Ca^2+^-free medium (containing 8.8 mmol/L MgCl_2_) was introduced 19 min before KCl. [^3^H]-D-Asp radioactivity was determined in each collected sample and in the superfused filters by Packard Tri-Carb 2111 TR liquid scintillation counter.

The amount of released [^3^H]-D-Asp in each collected sample was expressed as percentage of total synaptosomal radioactivity content at the beginning of the respective collection period (fractional rate×100).

Depolarization-evoked neurotransmitter overflow was estimated by subtracting the transmitter content of the two 3-min samples representing the basal release from that in the 6-min sample collected during and after the depolarization pulse. Appropriate controls were always ran in parallel.

### Preparation of tissue lysates

Retinas were lysed in ice-cold RIPA buffer (50 mM Tris-HCl (pH 8), 150 mM NaCl, 1 mM EDTA, 0.1% SDS, 1% IGEPAL and 0.5% sodium deoxicholate) containing protease inhibitor cocktails (code P8349; Sigma-Aldrich, Milan, Italy) and centrifuged for 15 min at 10.000 g at 4°C.

For experiments under native conditions retinas were lysed in ice-cold Native buffer (50 mM Tris-HCl (pH 8), 150 mM NaCl, 1% IGEPAL, 1 mM PMSF, 10% glycerol) containing protease (code P8349; Sigma-Aldrich, Milan, Italy) and phosphatase inhibitor cocktails (code 524625; Calbiochem, La Jolla, CA, USA). Lysates were centrifuged for 15 min, 10.000 g at 4 °C, and subjected to three cycles of freezing and thawing. In both cases, supernatants were collected and assayed for protein content by Bio-Rad DC protein assay kit (Bio-Rad Laboratories, Milan, Italy).

### Gel electrophoresis

#### One-dimensional gel electrophoresis: SDS or Native-PAGE

For western blotting analysis under reducing and denaturing conditions, equal amount (8–15 μg) of proteins were resolved by 10% sodium dodecyl sulfate (SDS)-polyacrilamide gel electrophoresis (PAGE). To analyze the protein of interest under native conditions 30 μg of total proteins were separated under non-denaturing conditions using a 5% stacking and a 6% separating native-polyacrylamide gel. After separation, proteins were transferred onto PVDF membranes (Immobilon-P, Sigma-Aldrich, Milan, Italy). For native samples, amido-black staining was used as loading control.

#### Two-dimensional gel electrophoresis: Native/SDS-PAGE

Gel lanes containing total proteins were cut out from Native-PAGE with a razor blade. Each lane was incubated with gentle agitation on a glass plate in a dissociating solution 1% (w/v) SDS and 1% (v/v) 2-mercaptoethanol for 1 h at RT and briefly rinsed with bi-distilled water. After three washes with SDS-PAGE electrophoresis buffer (25 mM Tris-HCl, 192 mM glycine and 0,1% (w/v) SDS; pH 8.3) the strip was rotated through 90° and placed onto SDS-PAGE using a 5% stacking and 10% separating gel. Native/SDS-PAGE containing total proteins was transferred onto PVDF membrane.

### Immunoblot analysis

Membranes were blocked with 5% non-fat milk in Tris-buffered saline containing 0.05% Tween 20 for 1 h at RT. Primary antibodies were incubated overnight at 4°C, followed by a horseradish peroxidase-conjugated secondary antibody for 1 h at RT. Protein bands were visualized with ECL Western Blotting Detection kit (ECL, Amersham Biosciences, GE Healthcare, Milan, Italy) and the chemiluminescence signal detected using X-ray films (Hyperfilm ECL, Amersham Biosciences). Autoradiographic films were scanned, digitalized at 300 dpi and band quantification was performed using ImageJ software (NIH, Bethesda, MD, USA). The following primary antibodies and dilutions were used: anti-GLT-1 1∶1000 (code 3838, Cell Signaling Technology, Beverly, MA, USA), anti-actin 1∶1000 (clone AC-40, Sigma-Aldrich, Milan, Italy), anti-β-tubulin 1∶80 000 (clone B-5-1-2, Sigma-Aldrich, Milan, Italy). Species-specific horseradish peroxidase-conjugated goat IgG (Pierce Biotechnology, Rockford, IL, USA) was used as secondary antibodies.

### Intravitreal administrations

Cycloheximide (CHX) (Sigma-Aldrich, Milan, Italy), a protein synthesis inhibitor [31], [32], was dissolved in sterile aqueous solution. Intravitreal injection was performed by puncturing the eye with a 23-gauge needle at the cornea–sclera junction and the drug was administered with a 5 µl Hamilton syringe (Bonaduz, GR, Switzerland). CHX (50 µg/3 µl/eye) or equal volume of control solution was administered at the end of the ischemia. The duration of the injection was 2 min in all instances. Animals were killed after 150 min of reperfusion and subjects with visible lens damage or vitreous hemorrhage were excluded from the study.

### Immunohistochemistry

Enucleated eyes were fixed in 2% paraformaldehyde (PFA) at 4°C for 10 min; after removal of the anterior segment, the posterior was fixed in 4% PFA for 60 min and cryopreserved in 30% sucrose overnight. Specimens were frozen in Optimal Cutting Temperature medium (Tissue-Tek, Sakura Finetek Europe, Alphen an den Rijn, The Netherlands), and 16-µm cryostat sections were cut, mounted onto Superfrost ultra plus glass slide (Menzel-Gläser, Braunschweig, Germany) and stored at −80°C until used. Retinal sections were washed in 0.1 M PBS (pH 7.4), permeabilized with 0.3% Triton for 45 min and blocked with 10% donkey serum (Sigma-Aldrich, Milan, Italy) at RT for 1 h. Slides were incubated overnight with rabbit anti-GLT-1 (1∶50, Cell Signaling Technology, Beverly, MA, USA) or mouse anti-GLAST (1∶300; code ab 41751, ABcam, Cambridge, UK). Immunofluorescence labeling was performed by incubation with anti-rabbit Alexa Fluor 488 (1∶250) or anti-mouse Alexa Fluor 488 (1∶500; Molecular Probes, Eugene, OR, USA) at RT for 1 h. Sections were mounted with Vectashield mounting media with DAPI to label the nuclei (Vector Laboratories, Burlingame, CA, USA). Image acquisition was performed using a confocal microscope (Leica TC-SP2 Confocal System; Leica Microsystems Srl, Milan, Italy).

### Statistical analysis

Data are given as mean ± S.E.M. of three to eight independent experiments and statistically evaluated for differences by Student's t-test or by one-way analysis of variance, followed by Tukey-Kramer test for multiple comparisons. A value of p<0.05 was considered to be statistically significant.
